# Innate Sensing of HIV-Infected Cells

**DOI:** 10.1371/journal.ppat.1001284

**Published:** 2011-02-17

**Authors:** Alice Lepelley, Stéphanie Louis, Marion Sourisseau, Helen K. W. Law, Julien Pothlichet, Clémentine Schilte, Laurence Chaperot, Joël Plumas, Richard E. Randall, Mustapha Si-Tahar, Fabrizio Mammano, Matthew L. Albert, Olivier Schwartz

**Affiliations:** 1 Institut Pasteur, Virus and Immunity Unit, URA CNRS 3015, Paris, France; 2 Center for Human Immunology, Department of Immunology, Institut Pasteur, Paris, France; 3 Institut Pasteur, Unité Défense Innée et Inflammation, Paris, France; 4 Institut Pasteur, Unité d'Immunobiologie des Cellules Dendritiques, Paris, France; 5 Université Joseph Fourier, La Tronche, France; 6 University of St. Andrews, St. Andrews, Scotland, United Kingdom; Fred Hutchinson Cancer Research Center, United States of America

## Abstract

Cell-free HIV-1 virions are poor stimulators of type I interferon (IFN) production. We examined here how HIV-infected cells are recognized by plasmacytoid dendritic cells (pDCs) and by other cells. We show that infected lymphocytes are more potent inducers of IFN than virions. There are target cell-type differences in the recognition of infected lymphocytes. In primary pDCs and pDC-like cells, recognition occurs in large part through TLR7, as demonstrated by the use of inhibitors and by TLR7 silencing. Donor cells expressing replication-defective viruses, carrying mutated reverse transcriptase, integrase or nucleocapsid proteins induced IFN production by target cells as potently as wild-type virus. In contrast, Env-deleted or fusion defective HIV-1 mutants were less efficient, suggesting that in addition to TLR7, cytoplasmic cellular sensors may also mediate sensing of infected cells. Furthermore, in a model of TLR7-negative cells, we demonstrate that the IRF3 pathway, through a process requiring access of incoming viral material to the cytoplasm, allows sensing of HIV-infected lymphocytes. Therefore, detection of HIV-infected lymphocytes occurs through both endosomal and cytoplasmic pathways. Characterization of the mechanisms of innate recognition of HIV-infected cells allows a better understanding of the pathogenic and exacerbated immunologic events associated with HIV infection.

## Author Summary

AIDS is characterized by a hyperactivation of the immune system. Innate and inflammatory responses, associated with an exacerbated production of cytokines like type I interferons (IFN) and of chemokines, deregulate the normal functioning of T lymphocytes and other cells. The events that trigger this inappropriate activation remain poorly understood. Plasmacytoid dendritic cells (pDCs) normally produce IFN when they encounter viruses. Here we examined how HIV-infected cells are recognized by pDCs, as well as by other immune and non-immune cells. We show that viruses transmitted via cell-to-cell contacts are more potent inducers of IFN than cell-free viral particles. In pDCs, recognition occurs in large part through TLR7, a cellular receptor detecting viral genetic materials after capture in intracellular vesicles. Donor cells expressing replication-defective viruses are also able to trigger IFN production by target cells. We further show that in TLR7-negative, non-hematopoietic cells an additional cytoplasmic pathway allows sensing of HIV-infected lymphocytes. Therefore, detection of HIV-infected lymphocytes occurs at different intracellular localizations, and does not require ongoing viral replication. Characterization of the mechanisms of innate HIV-1 recognition allows a better understanding of the pathology of HIV infection, and has consequences for the design of vaccine strategies.

## Introduction

HIV-1 infection is characterized by acute and chronic activation of the immune system [1], [2], [3]. Inappropriate innate and inflammatory responses trigger a cascade of events that deregulate T cell turnover, function and localization. B cells are polyclonally activated, the function of NK cells is impaired, circulating dendritic cells are decreased. This hyperactivation leads to a dysfunction of HIV-specific cellular and humoral immunity, and represents a driving force for CD4+ depletion, facilitation of viral replication, and AIDS progression. In natural hosts of SIV (such as African Green Monkeys and Sooty Mangabeys), the infection is generally non-pathogenic, despite high steady-state levels of plasma viral RNA. The most striking difference with pathogenic SIV infection in macaques is a lack of chronic activation in natural hosts [4]. Very early events, occurring within the first hours or days of viral exposure, likely influence the outcome of infection [5], [6]. The innate immune response, assessed by measuring type I interferons (IFN) and other cytokines, distinguishes non-pathogenic and pathogenic SIV infections. In natural hosts, type I IFN production is transient after viral inoculation [7], [8], and this may be related to low levels of Toll-Like Receptor (TLR) 7 and 9 signaling [6], or to an efficient down-regulation of acute type I IFN response [9]. In macaques, where SIV infection is pathogenic, an intense signaling occurs in the mucosae at the portal of viral entry, involving chemokines and a local accumulation of plasmacytoid dendritic cells (pDCs) producing type I IFN [5]. It is likely that similar early events are occurring during HIV-1 transmission and acute infection, which is often associated with a high production of IFN and pro-inflammatory cytokines [1]. However, the interactions between HIV-1, pDCs and other cells leading to cytokine production remain only partly characterized.

pDCs are a main component of the innate antiviral immune system. They are the major source of type I IFN and other antiviral cytokines, and contribute to the induction of adaptive immunity (reviewed in [10]). Viruses are detected by pDCs mainly through recognition of viral nucleic acids. These cells express TLR7 and 9 endosomal molecules, which recognize single stranded RNA (ssRNA) and unmethylated CpG-rich double stranded DNA (dsDNA), respectively. TLR7 and TLR9 stimulate IFN genes through IRF7 and inflammatory cytokines genes through NF-κB (reviewed in [11]). The number of circulating pDCs inversely correlates with plasma viral loads in HIV-infected individuals ([12] [13]). In culture experiments, pDCs produce IFN and other cytokines, and undergo phenotypic activation upon exposure to HIV-1 [14], [15], [16], [17]. IFN production by pDCs requires a high concentration of HIV-1 particles (>200 ng/ml Gag p24) [14], [15], [18]. The mechanisms of HIV recognition by innate receptors in pDCs are not fully understood. Both viral proteins and nucleic acids represent potential targets of recognition. Detection may occur after viral uptake, in an endosomal compartment, and/or after viral fusion, in the cytoplasm of target cells. Recombinant HIV Envelope glycoproteins (Env) induce type I IFN by PBMCs and pDCs, but also inhibit TLR9 activity [19], [20], [21]. In pDCs, HIV capture and endosomal degradation lead to viral RNA exposure and likely to TLR7 detection [15], [18]. The role of TLR7 was deduced from experiments using inhibitors of vesicular acidification and oligonucleotides targeting this receptor, but a direct demonstration of TLR7 involvement is still lacking. The ability of virion-associated Env to bind CD4 enhances IFN production by pDCs [22], but it is not clear whether binding itself, or subsequent Env-mediated fusion events mediate this phenomenon. After fusion, the viral RNA gains access to the cytoplasm, where reverse transcription occurs. This could lead to detection of nucleic acids by cytosolic sensors.

Besides pDCs, in most other cell types, viral nucleic acids may be recognized by various cytosolic pattern recognition receptors, including RLRs (RIG-I like Receptors). RLRs include DExD/H box-containing RNA helicase Retinoic acid Inducible Gene I (RIG-I) and Melanoma Differentiation Antigen 5 (MDA5). While RIG-I senses 5′-triphosphate RNA and short dsRNA (<1000 bp), MDA5 senses long dsRNA [23], [24], [25], [26], [27]. Some DNA viruses are also sensed by RIG-I, after transcription of viral DNA by RNA pol III [28] [29]. RIG-I and MDA5 stimulate IRF3 and NF-κB through the adaptor MAVS (also termed Cardif, Visa or IPS1), to produce IFN and inflammatory cytokines (reviewed in [11]). HIV recognition by non-pDCs is not fully understood. Whatever the cell type used, detection of cell-free HIV is generally considered sub-optimal. In macrophages and lymphocytes, TREX1, a host DNAse degrades HIV DNA generated during HIV infection, providing a mechanism for the virus to avoid detection by nucleic acid sensors [30]. However, CD4+ T cells from lymphoid tissues may sense incomplete viral reverse transcripts, and these abortive viral DNA products activate a host defense program eliciting proapoptotic and proinflammatory responses [31]. Monocytic-derived DCs are poorly sensitive to HIV infection, and produce low levels of IFN when encountering HIV [14]. When this resistance to productive infection is artificially circumvented, HIV-1 induces DC maturation and type I IFN production [32]. This cryptic process relies on the interaction of newly synthesized viral capsid with cellular cyclophilin [32].

Most of the studies regarding the sensing of HIV-1 have been performed with cell-free virions. However, PBMCs and pDCs efficiently sense HIV-infected CD4+ T lymphocytes [33], [34], but the cellular and molecular mechanisms involved are not characterized. Here, we examined how HIV-infected cells are recognized by pDCs, and by other cells. We show that infected lymphocytes are more potent inducers of IFN than free viral particles. We describe cell-type differences in the recognition of infected cells. In primary pDCs and pDC-like cells, recognition occurs mainly through TLR7. We provide evidence for additional pathways of sensing of infected cells. In a model of TLR7-negative non-immune cell, we show that the IRF3 pathway allows sensing of HIV-infected cells. Moreover, we show that cells expressing defective viruses are also recognized by PBMCs and may thus represent an underestimated source of hyperactivation of the immune system.

## Results

### Primary hematopoïetic cells efficiently sense HIV-infected cells

**Figure 1:**
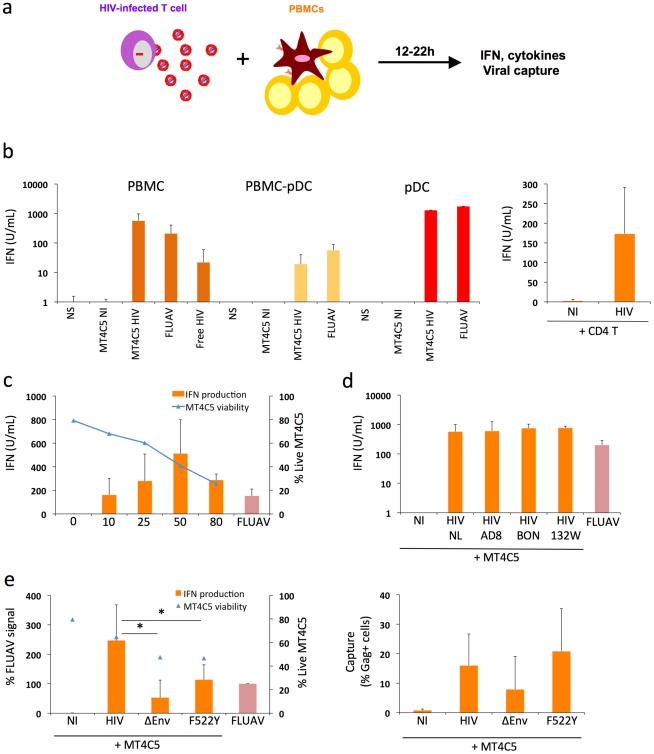
Sensing of HIV-infected lymphocytes by hematopoïetic cells. **a. Schematic representation of the experimental system.** HIV-1 infected T cells are mixed with PBMCs, and levels of IFN released in supernatants are measured 12-24h later. **b. IFN release in cocultures of HIV-infected T cells with PBMCs.** MT4C5 lymphoblastoid T cells (left panel) or primary CD4+ T lymphocytes (right panel) were used as donors. Whole PBMCs, PBMCs depleted of pDCs (PBMCs-pDCs), and purified pDCs where used as target cells. Targets were either left non stimulated (NS), cocultivated with non-infected (NI) or HIV-infected (NL4-3 strain) MT4C5 cells (MT4C5-HIV), exposed to cell-free HIV virions (free HIV) or Influenza virus (FLUAV). IFN production in supernatants was measured in a bioassay, after 12 h of culture. Donor to target cell ratio were 1∶2 for PBMCs and PBMCs-pDCs, and 2∶1 for pDCs. **c. Dose response analysis of IFN release.** Whole PBMCs were incubated with MT4C5 cells displaying increasing % of infected (Gag+) cells. IFN production was measured after 24 h of coculture (orange histrograms). The levels of living MT4C5 cells at the beginning of the coculture, determined by flow cytometry (forward and side scatters gating), are shown in blue. **d. Sensing of cells infected with various HIV isolates by PBMCs.** MT4C5, infected with NL4-3 (NL), NLAD8 (AD8) and with two primary isolates (BON and 132W) were cocultivated with PBMCs and IFN was measured 24 h later. With all isolates, about 25% of infected MT4C5 cells expressed Gag before coculture. **e. Role of HIV envelope glycoproteins.** MT4C5, infected with wild-type (HIV), Env-deleted (ΔEnv), or a non-fusogenic Env mutant (HIVF522Y), and with similar levels of Gag+ cells, were cocultivated with PBMCs and IFN was measured 12 h later (left panel). IFN levels are expressed as the % of the signal obtained with FLUAV. MT4C5 viability was similar with WT, ΔEnv, and F522Y HIV at the beginning of the coculture. The efficiency of HIV capture by PBMCs was assessed by measuring the % of Gag+ PBMCs by flow cytometry (right panel). Mean+sd of 3-5 independent experiments is shown, * p<0.05 (Kruskal-Wallis).

We first examined whether PBMCs and pDCs produce IFN when they encounter HIV-1. We compared the efficiency of recognition of infected cells and cell-free virions. We used as donor cells the MT4C5 lymphoid line (a derivative of MT4 cells expressing CCR5), which can be infected by CXCR4-tropic (X4) or CCR5-tropic (R5) HIV strains. Targets were fresh PBMCs isolated from healthy donors. Typically, pDCs, identified as BDCA4/BDCA2 double positive cells, represented 0.2 to 1 % of whole PBMCs (Fig. S1a). We also used as targets PBMCs depleted of pDCs (after removal of >80% of pDCs), or purified pDCs (corresponding to >85% of purity based on BDCA2/BDCA4+ double labeling) (Fig. S1a). Influenza virus (FLUAV, 40 HAU/mL), a well-characterized IFN inducer [35] was used as a positive control (Fig. 1b).

MT4C5 cells were infected with HIV-1 (X4 strain NL4-3). After two days of infection, when about 50% of the population was Gag+, MT4C5 cells were cocultivated with target cells. After 12 to 22 hours of coculture, IFN was measured in supernatants using a biological activity assay, and levels of HIV capture by targets were assessed by flow cytometry (the experimental protocol is outlined Fig. 1a). PBMCs were in parallel exposed to cell-free HIV (at 500 ng/mL Gag p24, a viral concentration corresponding to that reached in supernatants of infected MT4C5 cells during the coculture) (Fig. 1b). Strikingly, HIV-infected MT4C5 cells induced 10-100 times more IFN (mean of 600 U/ml) than cell-free HIV virions. Non-infected donor cells did not activate PBMCs (not shown). The supernatant of HIV-infected MT4C5 cells was not more active than purified viral particles (not shown).

When cocultivated with HIV-1 infected cells, PBMCs depleted of pDCs produced 10 times less IFN compared to total PBMCs (Fig. 1b). Conversely, purified pDCs produced huge amounts of IFN (up to 2,500 U/ml) (Fig. 1b). Thus, among circulating hematopoïetic cells, the early production of IFN induced by HIV-1 infected cells mainly originates from pDCs. To confirm this activation, we examined the induction of MxA, an IFN-stimulated gene (ISG) [36] in pDCs. Flow cytometry indicated that MxA was expressed by sorted pDCs cocultivated with HIV-infected MT4C5, or exposed to a high dose of cell-free HIV particles (1200 ng/ml p24), and not at a lower inoculum (600 ng/ml) (Fig. S1b). Therefore, HIV-infected cells promote IFN release and MxA expression by pDCs.

Other types of HIV-infected lymphocytes, like Jurkat cells (not shown) and primary CD4+ T cells (Fig. 1b), also promoted IFN production by PBMCs, demonstrating that this phenomenon is not an artifact due to the use of MT4C5 cells as donors. We also ruled out the possibility that the combined stimulus of HIV and mismatched HLA in heterologous cocultures of primary cells may induce a strong activation of IFN production. Direct infection of PHA-activated PBMCs with cell-free HIV lead to viral replication, with a peak of infected cells at day 3 post-infection (Fig. S2). IFN was also produced in supernatants, with a peak correlating with viral production. Therefore, the presence of HIV virions and infected cells triggers IFN release by PBMCs even in the absence of heterologous cells.

MT4C5 cells were then infected with various doses of HIV-1. After 2 days, the percentage of Gag+ cells varied from 10 to 80%, and cell viability inversely correlated with the extent of infection (Fig. 1c). After coculture with PBMCs, IFN production increased with the percentage of HIV-infected donor cells, with a peak at 50% of Gag+ donor cells. When 80% of donor cells were infected, viability of donor cells was low, accounting for the reduced IFN levels released by PBMCs (Fig. 1c). We then induced apoptosis of MT4C5 cells by treatment with Etoposide or Camptotecin (up to 40% of apoptotic cells, not shown). Apoptotic MT4C5 cells did not elicit IFN release by primary PBMCs (not shown). This suggests that the apoptotic events associated with HIV infection are not responsible for the activation of pDCs. The experiments described above were performed at a ratio of 1 donor for 2 target PBMCs (1∶2). At a lower ratio (1∶5 to 1∶10), levels of IFN diminished progressively, likely reflecting the decreased capture of viral materials by target cells (not shown).

We also examined if infected MT4C5 cells may have produced very low levels of type I IFN that could trigger a secondary response by pDCs. However, we could not detect IFN production by HIV-infected MT4C5 cells, using three sensitive assays: measurement of type I IFN in supernatants with the Luminex technology, with a biological activity assay, and by quantifying type I IFN mRNA in infected cells by qPCR (not shown). Similarly, HIV-infected MT4C5 cells did not induce type I IFN when mixed with uninfected MT4C5 cells (not shown). This lack of type I IFN production by HIV-infected MT4C5 cells, as well as by cocultures of infected and non-infected MT4C5 cells will require further investigation, and is in line with a known disruption of innate antiviral signaling and immune defenses within infected cells [37]. Moreover, the addition of uninfected MT4 cells did not enhance the detection of cell-free HIV by PBMCs, when assessed at 12 h of incubation (not shown). At later time points, MT4C5 cells became infected and activated PBMCs (not shown).

We next tested the ability of various HIV-1 strains to induce IFN production. MT4C5 cells, infected with three R5 isolates (the reference strain NLAD8 and two primary viruses BON and 132W [38]) induced similar levels of IFN in PBMC targets (Fig. 1d), indicating that this phenomenon is not restricted to laboratory-adapted viruses.

It is noteworthy that there were some donor-to-donor variations in the levels of IFN response to HIV-infected cells. All of the 16 different donors tested produced type I IFN after a 12 h-coculture with HIV-infected MT4C5 cells, with a range of 600±400 U/ml.

Together, the results indicate that induction of IFN in PBMCs correlates with the level of infection but is also dependent on the viability of donor cells. A high rate of cellular mortality does not enhance IFN production by PBMCs. Various laboratory-adapted and primary HIV isolates induce IFN.

### Recognition of HIV-infected cells is enhanced by functional viral envelope glycoproteins

In pDCs, the main cellular receptor mediating HIV binding is the CD4 molecule. These cells, unlike myeloid DCs, express neither DC-SIGN nor other lectins binding Envelope glycoproteins (Env) with high affinity [39]. Accordingly, antibodies against CD4 or Env inhibit activation of pDCs by HIV virions [15], [22]. We extended this observation, and asked whether Env is required for the recognition of HIV-infected cells by PBMCs. We used as donors MT4C5 cells producing an Env-deleted (ΔEnv) or a fusion-defective HIV (F522Y mutant) [40]. F522Y carries a point mutation in Env, which abrogate fusion but retains CD4 binding ability. Since these viruses are not infectious, MT4C5 cells were infected with ΔEnv or F522Y particles pseudotyped with a VSV-G envelope, to ensure viral entry. ΔEnv-infected MT4C5 barely elicited IFN release by PBMCs, and there was a two-fold decrease in the potency of F522Y-infected cells to activate PBMCs, when compared to WT HIV (Fig. 1e). The level of viral binding or capture by PBMCs was assessed by measuring the % of Gag+ target cells by flow cytometry, after 12 hours of coculture (Fig. 1e). WT and F522Y HIV were similarly uptaken by target PBMCs, whereas ΔEnv, as expected, was less captured. Therefore, the fusogenic activity of Env increases recognition of infected cells by PBMCs. This suggests that recognition takes place mainly within endosomes, but also once the virus has entered the cytoplasm of target cells.

### Pathways of recognition of HIV-infected cells by PBMCs

**Figure 2:**
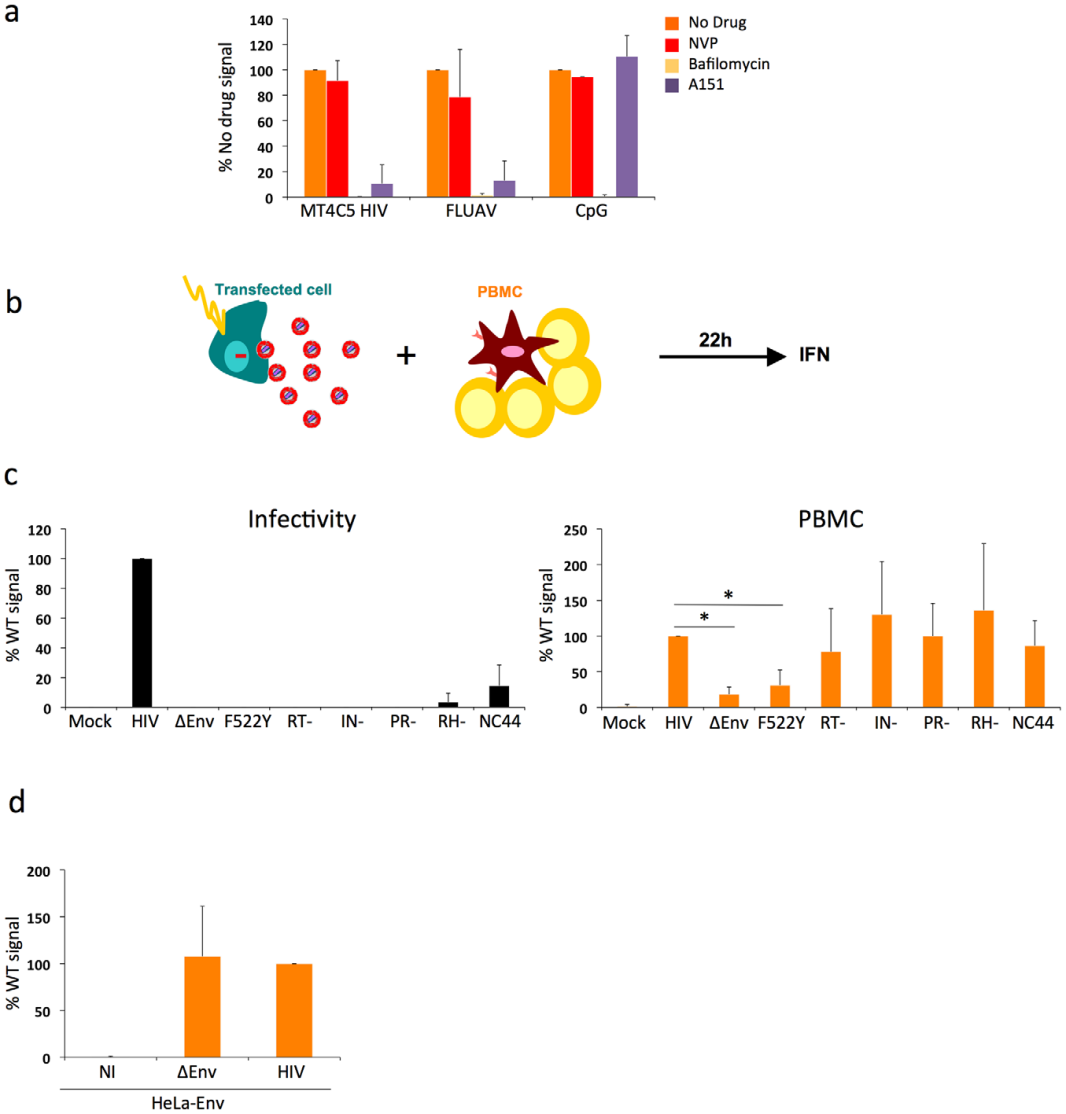
Pathways of recognition of HIV-infected cells by hematopoïetic cells. **a. Effect of drugs targeting endosomal TLR signaling or HIV reverse transcription in PBMCs.** PBMCs were cocultivated with HIV-infected MT4C5 cells, exposed to FLUAV, or to CpG (a TLR9 agonist) during 12 h, in the absence or presence of the indicated drugs. NVP (25 µM) is a reverse transcriptase inhibitor. Bafilomycin A1 (Bafilo, 125 nM) is an inhibitor of vesicular acidification. A151 (5 µg/mL) is a TLR antagonist. Nevirapin (NVP 25 µM) is a reverse transcriptase inhibitor. IFN levels are expressed as the % of the signal obtained without drugs. Mean+sd of 3 independent experiments is shown. *p<0.05 (Kruskal-Wallis). **b-d. Viral proteins required for the recognition of HIV-expressing cells by PBMCs. b. Schematic representation of the experimental system.** HeLa cells are transfected with various HIV mutants, cocultivated with PBMCs, and levels of IFN released in supernatants are measured 22 h later. **c. Defective viruses trigger IFN production by PBMCs.** HeLa cells were transfected with the indicated proviral mutants. Supernatants were harvested and analyzed for the presence of infectious virus, after normalization for Gag p24 levels. Env-deleted (ΔEnv), non-fusogenic Env (HIV F522Y), reverse transcriptase (RT-), integrase (IN), protease (PR), Rnase H (RH), and nucleocapsid (NC44) mutants are not or poorly infectious (left panel). Levels of IFN in supernatants, after coculture of tranfected HeLa cells with PBMCs. IFN levels are expressed as a percentage of the signal obtained with WT HIV (right panel). **d. Expression of Env alone does not trigger IFN production.** HeLa-Env cells (stably expressing a functional Env glycoprotein complex) were either not infected (NI) or infected with ΔEnv or WT viruses (pseudotyped with VSV), then processed and analyzed as in c. c-d: Mean+sd of 2-3 independent experiments is shown. *p<0.05 (Kruskal-Wallis).

To determine the mechanisms by which PBMCs sense HIV-infected lymphocytes, we first evaluated the effect of Bafilomycin A1, an inhibitor of vesicular acidification. As controls we utilized FLUAV and CpG, stimuli for TLR7 and TLR9, respectively [35] [41]. Bafilomycin A1 (125 nM, a concentration that did not detectably affected cell viability at 24 h, not shown) inhibited HIV recognition (Fig. 2a), consistently with the requirement for an acidic endosome and viral degradation in order to achieve TLR signaling [15], [42], [43]. Of note, Bafilomycin A1 at 25 nM also inhibited HIV recognition (not shown). FLUAV and CpG stimulation were strongly inhibited by Bafilomycin A1, as expected. We also tested the effect of A151, an oligonucleotide described as a TLR antagonist, (inhibiting TLR7 and, to a lower extent, TLR9) [15], [43]. A151 (5 µg/mL) inhibited IFN production by PBMCs cocultivated with HIV-infected MT4C5 cells (Fig. 2a). At 5 µg/ml, A151 inihibited FLUAV, but not CpG stimulation of PBMCs. At a higher concentration (20 µg/ml), A151 partly inhibited CpG stimulation (not shown), confirming that this compound may antagonize both TLR7 and TLR9. Altogether, these results suggest that detection of HIV-infected cells by pDCs and by PBMCs requires an acidic environment and is in large part mediated by endosomal TLRs.

We thus asked whether viral reverse transcription plays a role in this detection. Nevirapine, a reverse transcriptase (RT) inhibitor, was pre-added on infected donor cells and maintained during the coculture. With Nevirapine, the levels of Gag in targets cultivated for 12 h with HIV-infected MT4 donor cells corresponded to those observed with F522Y. At 36 h, Nevirapine decreased the appearance of newly synthesized Gag proteins in PBMCs (not shown). This suggested that Nevirapine did not prevent viral capture by PBMCs but decreased at least partly subsequent productive infection. Interestingly, Nevirapine did not inhibit IFN production by PBMCs (Fig. 2a). Viral reverse transcription, and hence neo-synthesis of viral DNA and further steps of the viral life cycle, are thus probably not necessary to elicit IFN release.

### Additional TLR-independent pathways involved in HIV activation of PBMCs

While our data supports the engagement of TLRs on pDCs, the decreased detection of envelope-deficient viruses suggested that other sensors might also be implicated. That Nevirapine did not inhibit IFN production suggests that reverse transcription and productive infection are not required to activate targets. We used a panel of viral mutants to document this observation further, and to determine which step of the viral life cycle triggers immune recognition of infected cells. We assessed the activity of viruses carrying mutations in viral Reverse Transcriptase (RT), Integrase (IN), RNAse H (RH), Protease (PR) enzymes. These defective viruses are arrested at different steps of the viral cycle following fusion and entry, leading to production of different types of viral nucleic species in target cells. With the HIV RT- mutant, recipient cells will only mainly “see” viral RNA genome. The HIV IN- mutant synthesizes proviral DNA that will not integrate into the host genome and will accumulate in the cytoplasm of target cells. The RH- mutant is defective for the ribonuclease H activity, which is necessary for viral RNA degradation during reverse transcription, thus leading to a premature arrest of viral DNA synthesis [44]. The PR- mutant is not infectious, since viral enzymes and Gag and Gag-Pol precursors are not processed. We also evaluated the efficacy of a virus mutated in the nucleocapsid (NC44 mutant) [44]. This mutant loses about 90% infectivity, reverse transcription prematurely occurs in producer cells and in viral particles, which then contain abnormally high levels of proviral DNA.

Since these mutants do not infect lymphoid cells, we transfected the corresponding proviral constructs in HeLa cells (Fig. 2b). We also transfected WT, ΔEnv and F522Y mutants, to study the role of viral access to the cytoplasm when donors are HeLa cells. Upon transfection with this panel of viral mutants, HeLa cells displayed similar levels of Gag+ cells (10-20%) and Gag p24 release (10–50 ng/ml) in supernatants (not shown). As expected, these mutants were not (ΔEnv and F522Y, RT-, IN-, RH-, PR-) or very poorly (NC44) infectious when tested in a single cycle infectivity assay (Fig. 2c). We then cocultivated HeLa cells with PBMCs for 22 h. HeLa cells expressing WT HIV, and not control cells (transfected with an irrelevant plasmid), induced IFN production by PBMCs (up to 60 U/mL, not shown). This range of IFN is much lower than that induced by HIV-infected lymphocytes (Fig. 1). This reduction could be in part due to the lower production of HIV by transfected HeLa cells, when compared to infected MT4C5 cells. Interestingly, in this experimental system, ΔEnv- and F522Y-expressing cells did not stimulate target cells. In contrast, all other mutants elicited IFN production by PBMCs (Fig. 2c).

HeLa cells stably expressing a functional Env at their surface (HeLa-Env cells) [45] did not elicit IFN production by PBMCs (Fig. 2d). HeLa-Env cells also express Tat [45]. Therefore, the presence of Tat in cocultures of Env+ cells and target PBMCs is not sufficient to trigger type I IFN production. As expected, infection of HeLa-Env cells with ΔEnv or WT HIV proviruses restored activation (Fig. 2d). We also examined the effect of a transient contact between donor and target cells. We harvested target PBMCs after a 2 h-coculture with HIV-expressing HeLa cells. This short contact was sufficient to induce type I IFN production in target cells, albeit at lower levels than a continuous coculture (not shown). Thus, activation of PBMCs in this system does not require a sustained viral production by donor cells.

In sum, in this experimental setting characterized by a low level of viral production by donor HeLa cells, the step of viral fusion significantly enhances activation of target PBMCs. Expression of Env alone is not sufficient to mediate sensing. Reverse transcription, as well as later steps of the viral cycle do not enhance IFN production, at least during the first 24 h of coculture. Moreover, PBMCs efficiently sense cells producing defective viruses.

### Sensing of HIV-infected cells by the Gen2.2 pDC-like cell line

**Figure 3:**
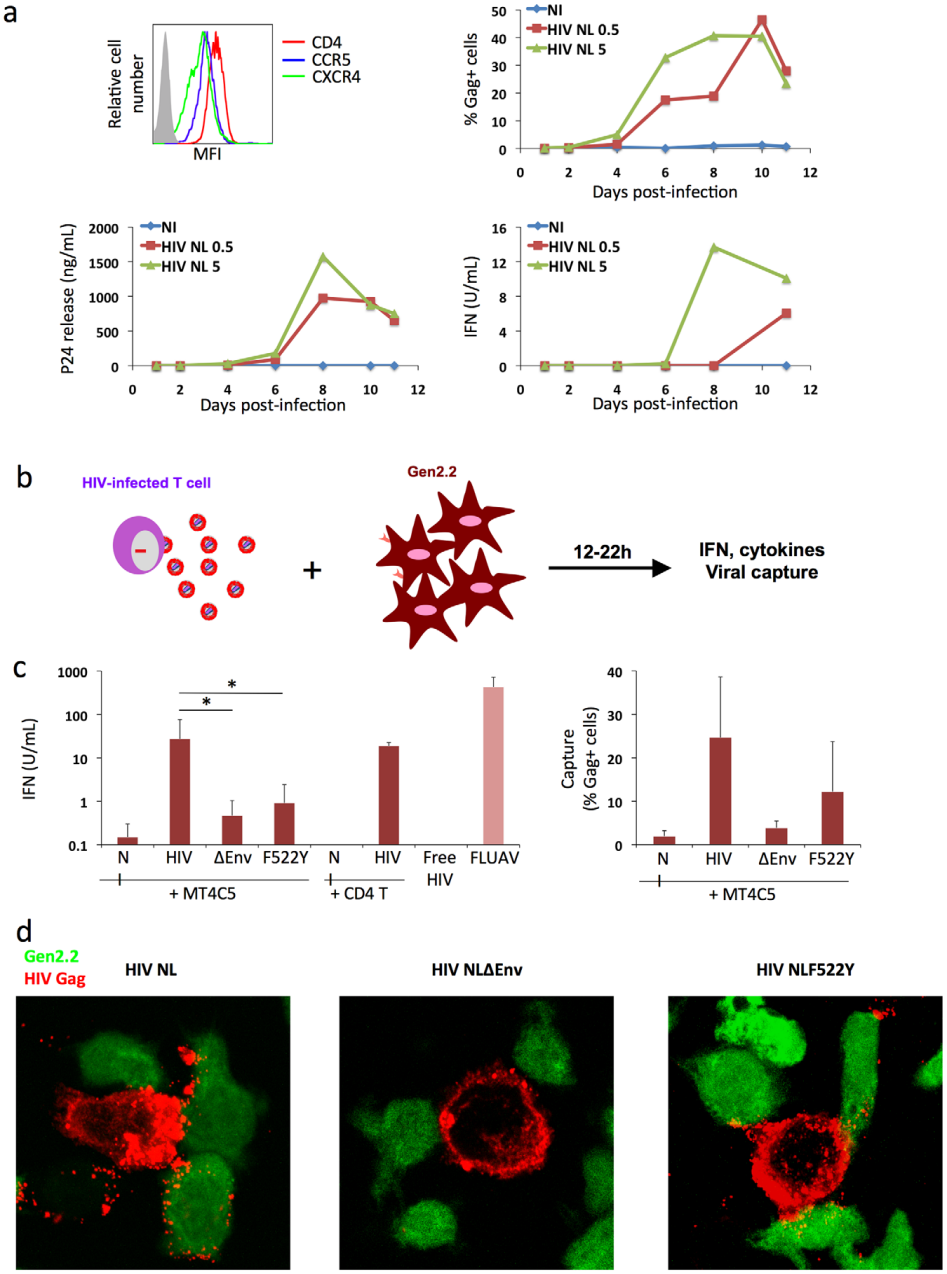
Recognition of HIV-infected cells by the Gen2.2 plasmacytoid-like cell line. **a. Gen2.2 cells express HIV receptors and are sensitive to HIV infection.** Surface expression levels of CD4, CXCR4 and CCR5 are shown on the upper left panel. Gen2.2 cells were then exposed to HIV particles, NL4-3 strain, at two doses (0.5 and 5 ng/0.1 ml Gag p24/10^6^ cells). Viral replication was assessed by following the appearance of Gag+ cells (upper right panel) and Gag p24 release in supernatants (lower left panel). IFN production was measured in the same supernatants (lower right panels). Data are representative of 3 independent experiments. **b. Schematic representation of the coculture system.** HIV-1 infected T cells are mixed with Gen2.2 cells and levels of IFN released in supernatants are measured 12–22 h later. **c. Sensing of HIV-infected cells by Gen2.2 and role of viral envelope glycoproteins.** MT4C5, infected with wild-type (HIV), Env-deleted (ΔEnv), or a non-fusogenic Env mutant (HIVF522Y), and with similar levels of Gag+ cells, were cocultivated with PBMCs and IFN was measured 22 h later (left panel). When stated, HIV-infected primary CD4+ cells were used as donors, instead of MT4C5 cells. Cell-free HIV virions (500 ng/ml p24) do not stimulate Gen2.2 cells. The efficiency of HIV capture by Gen2.2 was assessed by measuring the % of Gag+ cells by flow cytometry (right panel). Mean+sd of 10 independent experiments is shown, *p<0.05 (Kruskal-Wallis). **d. Visualization of HIV capture by Gen2.2 cells.** Gen2.2 cells were labeled with CFSE (green) and cocultured with MT4C5 infected with wild-type (HIV), ΔEnv and F522Y mutants. Cells were harvested after 1 h and stained for HIV Gag (red). Representative fields are shown.

We sought to use the Gen2.2 pDC-like cell line to dissect the pathways of recognition of HIV-infected cells. This cell line was derived from a patient with pDC leukemia, and possesses phenotypic and functional features of primary pDCs [46]. Gen2.2 cells express TLR7, TLR9 and other pDC markers. They produce IFN when exposed to FLUAV or other TLR agonists, although at lower levels than primary pDCs. Gen2.2 mature upon incubation with very high concentration of HIV particles [16]. These cells represent a useful tool as they can be transduced with lentiviral vectors encoding for shRNAs [47]. We first examined whether Gen2.2 cells are sensitive to HIV replication. Like primary pDCs [48], these cells express the HIV receptors CD4, CXCR4 and CCR5 (Fig. 3a). Gen2.2 cells efficiently replicated HIV, as assessed by measuring the appearance of Gag+ cells and viral release by Gag p24 ELISA in supernatants at different time points after infection (Fig. 3a). Viral release was particularly high, reaching 1600 ng/ml Gag p24 at day 8 pi. IFN was also detected in supernatants, but only at late time points (day 8–10 pi). IFN release reached 15 U/ml, when about 30-40 % of Gen2.2 where Gag+ (Fig. 3a). Gen2.2 cells thus replicate HIV-1 and produce IFN upon infection.

**Figure 4:**
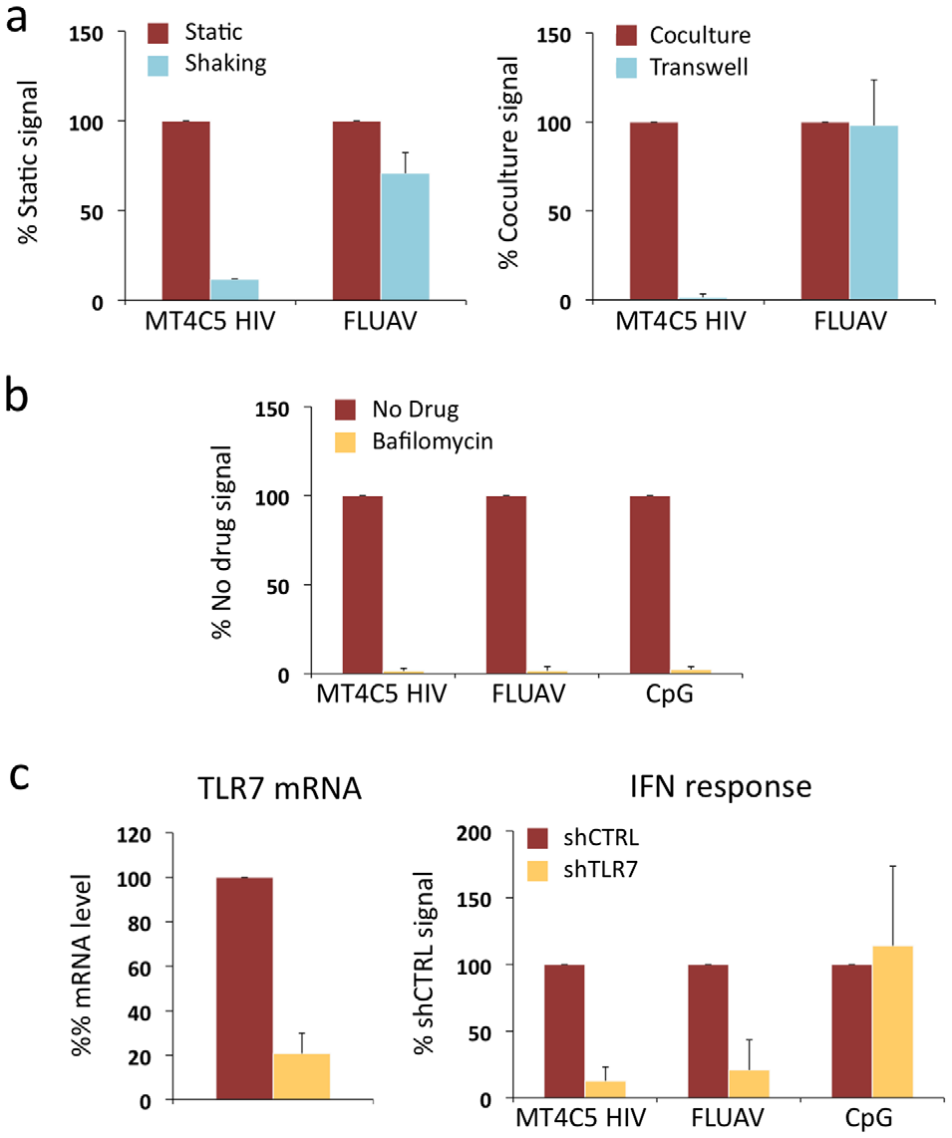
Role of cell contacts and of TLR7 on recognition of HIV-infected cells by Gen2.2 cells. a. Activation of Gen2.2 requires a direct contact with HIV-infected cells. Shaking cocultures of Gen2.2 and HIV-1-infected MT4C5 (left panel), or separating infected MT4C5 cells and target Gen2.2 in a transwell chamber (right panel) impairs IFN production, whereas recognition of FLUAV by Gen2.2 is not affected. Mean+sd of 3 independent experiments is shown **b. Inhibition of IFN production by Bafilomycin A1**. Gen2.2 cells were cocultivated with HIV-infected MT4C5 cells, exposed to FLUAV, or to CpG (a TLR9 agonist) during 24 h, in the absence or presence of Bafilomycin A1 (Bafilo 125 nM). IFN levels are expressed as the % of the signal obtained without drugs. **c. Role of TLR7.** Gen2.2 cells were transduced with lentiviral vectors expressing shRNAs against TLR7 (shTLR7) or an irrelevant target (shCTRL). Levels of TLR7 mRNA in transduced cells were measured by RT-PCR. Data are normalized to GAPDH mRNA and expressed as relative levels of mRNA compared to shCTRL cells. Mean+sd of 3 independent experiments are shown (left panel). IFN production in TLR7-silenced and control Gen2.2 cells, cocultivated with HIV-infected MT4C5 or stimulated with FLUAV or CpG for 40 h. IFN levels are expressed as a percentage of the signal obtained with shCTRL Gen2.2 cells.

Next, we examined how Gen2.2 cells reacted when encountering HIV-infected T cells. To this end, infected MT4C5 were mixed with Gen2.2, and IFN levels were measured at 12 or 24 h of coculture. IFN was barely detected at 12 h (not shown). At 24 h, about 25 U/ml IFN were released in supernatants (Fig. 3c), which is 10–30 fold less than in primary pDCs. FLUAV potently activated Gen2.2 cells, whereas cell-free HIV particles (500 ng/ml Gag p24) elicited no IFN release (Fig. 3c). We reported that a gentle shaking of cocultures of infected cells and target cells inhibits direct cell-to-cell viral spread [49]. Shaking cocultures of HIV-infected MT4C5 cells and Gen 2.2 cells strongly decreased IFN production, whereas induction by FLUAV was not affected (Fig. 4a). Furthermore, IFN levels were below detection levels when infected donors cells and target Gen2.2 recipients were separated by a Transwell chamber (Fig. 4a). This strongly suggested that a direct contact between HIV-infected lymphocytes and Gen 2.2 cells is required to mediate recognition. Confirming our observation made with primary pDCs, optimal recognition of infected cells required a functional Env, as evidenced by the low levels of IFN (<1 U/ml) induced by ΔEnv- and F522Y-infected MT4C5 cells. HIV capture by Gen2.2 cells was assessed by flow cytometry after 12 h of coculture (Fig. 3c). WT and, to a slightly lower extent, F522Y HIV were efficiently captured, whereas very few Gen2.2 cells were Gag+ after contact with ΔEnv-expressing lymphocytes. These differences in HIV capture by Gen2.2 cells were already visible after 1 h of coculture, as evidenced by immunofluorescence staining (Fig. 3d). WT and F522Y-infected cells formed virological synapses or polysynapse-like structures [50] with Gen2.2 cells, and patches of viral (Gag+) material were readily transferred to targets. Of note, coculture of Gen2.2 with HIV-1 infected primary CD4+ lymphocytes activated Gen2.2 as efficiently as MT4C5 cells (Fig. 3c).

Therefore, although they produce less IFN, Gen2.2 cells behave like primary pDCs and efficiently sense HIV-infected cells. As with primary cells, Bafilomycin A1 (125 nM) inhibited HIV recognition (Fig. 4a). We next assessed the involvement of TLR7 in the sensing of HIV-infected cells. TLR7 expression was silenced by transduction of Gen2.2 cells with a lentiviral vector coding for an anti-TLR7 shRNA. The vector also expressed a puromycin-resistance gene, allowing selection of a population of transduced cells (termed Gen2.2-shTLR7). Silencing decreased TLR7 mRNA levels by 80%, when compared to a control shRNA (Fig. 4b). Gen2.2-shTLR7 cells produced about 10-fold less IFN than control cells, when cocultivated with HIV-infected MT4C5 cells (Fig. 4b). Response to FLUAV was impaired when TLR7 was downregulated, whereas CpG stimulation was not affected. These experiments directly demonstrate that TLR7 is a cellular receptor mediating recognition of HIV-infected cells by pDCs.

### Sensing of HIV-infected cells by TLR7-negative epithelial-like cells

**Figure 5:**
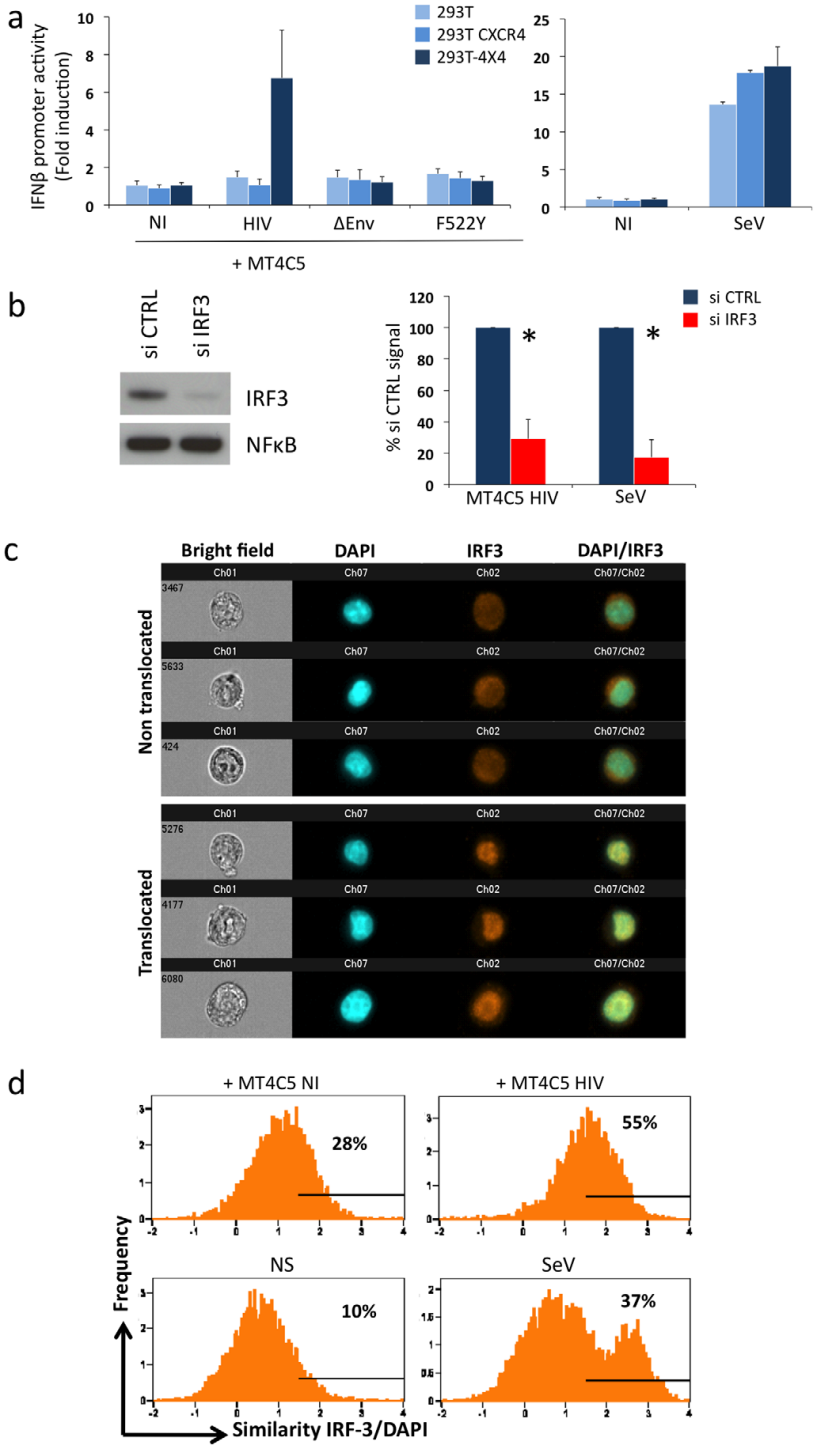
Sensing of HIV-infected lymphocytes by 293T-derived epithelial cells. **a. Role of CD4 and CXCR4 in 293T cells.** Activity of the IFNβ promoter in parental 293T cells, and in 293T cells expressing CXCR4 (293T CXCR4), or CD4 and CXCR4 (293T-4X4). These cells were transfected with IFNβ-luciferase reporter plasmid, and cocultivated for 16 h with MT4C5 cells expressing wild-type, ΔEnv, or F552Y HIV. The fold induction of luciferase activity, compared to non-stimulated cells is shown. The paramyxovirus Sendai virus (SeV) was used as a positive control (right panel). **b. Role of IRF3.** 293T-4X4 cells were transfected with an irrelevant (CTRL) or an anti IRF3 siRNA. 48 h later, cells were cocultivated with HIV-infected MT4C5 cells and assayed for IFN-β promoter activity. Left panel: IRF3 and NFkB levels, assessed by western blot, in control (CTRL) and silenced 293T-4X4 cells. Right panel: IFN-β promoter activity is expressed as a percentage of the signal obtained with control cells (CTRL). SeV, which signals through IRF3, was used as a control. Mean+sd of 3 independent experiments is shown. *p<0.05 (Kruskal-Wallis). **c–d. Automated quantification of IRF3 nuclear translocation.** 293T-4X4 cells were cocultivated with non-infected (NI) or HIV-infected Far-red dye stained MT4C5 cells, exposed to SeV, or left non-stimulated (NS) for 16 h. Cells were then stained with anti-IRF3 Abs and nuclei were visualized with DAPI. Automated quantification of IRF3 nuclear translocation on gated 293T-4X4 (Far-red dye negative) cells was performed using the ImageStream technology. c. Representative images of Bright field, Dapi (blue) IRF3 (green), DAPI/IRF3 composite images, for cells with either a diffuse intracellular (non translocated, low similiarity value) or a nuclear localization (translocated, high similarity value) are shown. d. 293T-4X4 cells with nuclear-localized IRF3 have higher similarity values, because the aspect of IRF3 and DAPI staining is similar. The percentage of cells with similarity values above an arbitrary value of 1.5 (where most, if not all of the cells fall in the translocated category) is indicated on each histogram. Data are representative of 4 independent experiments.

The experiments with fusion-defective viruses described above implicated cytosolic receptors in HIV recognition. We asked whether HIV-infected lymphocytes can be detected by additional, TLR7 independent cellular pathways. To this end, we performed cocultures with 293T cells as targets, as these latter do not express TLR7, TLR9 and TLR3 [51] and are widely used to dissect pathways of IFN signaling. We confirmed that 293T cells do not express detectable levels of TLR7 mRNA by real-time PCR, and that they are refractory to stimulation by the TLR7/8 agonist Gardiquimod (not shown). Next, we evaluated whether HIV-1 infected lymphocytes could activate in 293T cells a luciferase reporter that is under the control of the IFNβ promoter (IFNβ-luc). This technique is widely used to assess the IFNβ pathway in 293T cells [52], [53], [54], [55], because the levels of IFN released in the supernatants are below detection limits (not shown). Importantly, there was no induction of luciferase over background levels, upon coculture of HIV-infected MT4C5 cells with IFNβ-luc transfected 293T cells (Fig. 5a). 293T cells lack HIV receptors CXCR4 and CD4. We generated 293T cells expressing either CXCR4 (293T-CXCR4) or both CD4 and CXCR4 (293T-4X4 cells). In contrast to parental cells and to 293T-CXCR4 cells, 293T-4X4 cells activated the IFNβ promoter, when mixed with MT4C5 cells infected with wild-type HIV (7 fold increase, when compared to non-infected cells). Noteworthy, MT4C5 cells expressing either ΔEnv or the non-fusogenic F522Y strain failed to elicit luciferase activity (Fig. 5a). As a positive control, we used Sendai virus (SeV), a parainfluenza virus that activates IFNβ in 293T cells through the RIG-I/IRF3 pathway [52], [53], [54]. Moreover, free HIV virions (even at high concentrations) did not activate 293T-4X4 (not shown). Altogether, these results indicated that viral fusion activates the IFNβ promoter in the absence of TLR7. Detection of HIV-infected cells is more efficient than that of virions also in this experimental system.

We characterized further the TLR7-independent pathways that mediate recognition of HIV-infected cells. The binding of some viral RNA to RIG-I and MDA5 helicases leads to conformational changes that expose their CARD-like domains, induces binding to MAVS, and down-stream signaling through IRF3 [53], [56], [57], [58]. We thus silenced IRF3 expression in 293T-4X4 cells by siRNA transfection. Western blot analysis confirmed that IRF3 was barely detectable in silenced 293T-4X4 cells, whereas NFkB was not affected (Fig. 5b). Interestingly, IRF3 was required for the activation of the IFNβ promoter in 293T-4X4 cells cocultivated with HIV-infected cells (75% decrease in IRF3-silenced cells) or exposed to SeV (90% decrease) (Fig. 5b). We then used the ImageStream cytometer that combines flow cytometry with intracellular localization of fluorescent proteins, to examine nuclear translocation of IRF3. This technique allows a quantitative assessment of the localization of transcription factors in a large number of cells (a few thousands per sample) [59], [60], [61]. 293T-4X4 cells were cocultivated with non-infected or HIV-infected MT4C5 cells for 16 hours. MT4C5 cells were first stained with Far Red dye, to distinguish them from 293T-4X4 cells. Fixed cells were then stained with anti-IRF3 antibodies, and nuclei were visualized with DAPI. Non-stimulated or SeV-exposed cells were used as negative and positive controls, respectively. Images were then captured in five chanels: darkfield, brightfield, Far Red, DAPI, IRF3. Representative images of gated 293T-4X4 cells displaying either a diffuse intracellular localization (non translocated) or a colocalization with DAPI (translocated) are depicted Fig. 5c. A quantitative analysis based on the similarity of IRF3 and DAPI stainings revealed a 2-fold increase when 293T-4X4 were incubated with HIV-infected cells (55% vs 28% for cocultures of HIV-infected and non infected MT4C5 cells, respectively, Fig. 5d). As expected, with SeV, there was a 3-4-fold increase in the similarity score. Therefore, contact with HIV-infected cells induces nuclear translocation of IRF3 in 293T-4X4 cells.

Altogether, these experiments indicate that in the absence of endogenous TLR7 a pathway involving IRF3 triggers recognition of HIV-infected cells.

## Discussion

Our study delineates some features of HIV innate recognition. Cell-free HIV particles are generally considered as poor inducers of type I IFN [14], [30], [32]. We show here that HIV-infected cells are much more potent stimulators of IFN than free viral particles, confirming and extending previous findings [34]. Various cell types may recognize HIV-infected lymphocytes. Among PBMCs, pDCs are the main cells detecting HIV-infected cells, since their removal strongly decreases IFN release. TLR7 senses HIV components, but additional pathways are operative. Viral fusion is an important step promoting HIV detection, whereas reverse transcription and subsequent events of the viral life cycle are not necessary to activate an immune response. Using a model of cells lacking TLR7, we demonstrate that the IRF3 pathway participates in HIV recognition. Therefore, the host innate response to HIV involves the integration of multiple sensor pathways.

Why detection of HIV-infected cells is more efficient than that of cell-free virions? A contact with infected cells promotes a massive and rapid transfer of viral material to target cells [49], as visualized here in pDCs by immunofluorescence and flow cytometry. We show here, by shaking cocultures and by using transwell chambers, that a direct contact between infected cells and recipients is required to trigger high levels of IFN release by these latter. Various modes of cell-to-cell HIV transfer have been reported in culture, including synapses, polysynapses, filopodial bridges and nanotube-like structures [62]. HIV dissemination involves viral endocytosis in target cells [63], [64]. Whatever the mode of cell-to-cell transfer, the viral influx is quantitatively more important than capture of cell-free viral particles and may explain in part the potent induction of IFN production. Furthermore, infected cells may produce additional cellular or viral components that are not found in virions, which may enhance the activation of target cells.

Detection of HIV-infected cells by pDCs and PBMCs requires functional viral envelope glycoproteins. Envelope-deleted viruses are not or poorly recognized, mainly because target cells capture limited amounts of viral material in the absence of Env glycoproteins. When produced in MT4C5 cells, the fusion-defective envelope mutant (F522Y) is efficiently captured by PBMCs, but its ability to stimulate pDCs is reduced. This raises interesting mechanistic insights. F522Y HIV is normally up-taken in the endosomal compartment but further access to the cytosol is compromised [65]. Thus, there are multiple sites of recognition of incoming material from HIV-infected cells. In endosomes, TLRs may detect incoming HIV, as illustrated by the inhibitory effect of Bafilmoycin A1 and of TLR antagonists. After fusion, additional mechanisms of detection may enhance IFN production.

Several lines of evidence suggest that viral replication and productive infection of PBMCs are not necessary to trigger a response to infected cells. Nevirapine, a reverse transcriptase inhibitor, does not inhibit IFN release by PBMCs. However, it is possible that Nevirapine does not fully inhibit viral DNA synthesis in coculture systems of viral cell-to-cell spread. As incomplete viral DNA products represent a source of cytoplasmic nucleic acids that activate an innate immune response [31], we examined the activity of a series of viral mutants, arrested at different steps of the viral cycle. We show that HeLa cells expressing defective viruses, inactivated either in their reverse transcriptase, RNAse H, integrase, protease or nucleocapsid, stimulate IFN release in PBMC cocultures as efficiently as WT HIV. In this coculture system, in which HeLa cells produce relatively low levels of virus, viral fusion activity enhances recognition, as demonstrated with ΔEnv and F522Y mutants that triggered low levels of IFN production. Whether virus-cell or cell-cell fusion, or a combination of both, cause PBMCs activation will require further investigation. Cell-cell fusion is a feature of many viral infections, including HIV. The resulting syncytia may represent a structure producing high levels of IFN, as reported for Measles-induced giant cells [66]. Noteworthy, Env expression alone was not sufficient to activate PBMCs. It will be worth assessing the stimulating effect of donor cells producing particles devoid of HIV RNA.

The majority of HIV particles produced by an infected cell are defective or lead to abortive infection [67] [31], [68]. Defective viruses are generated by reverse transcriptase errors, editing activity of APOBEC proteins, and by various other mechanisms. Our results suggest that cells producing non-replicating viruses, if retaining fusion potential, may represent an underestimated source of innate immune activators. Our results also confirm that abortive infection may be sensed in target cells [31], thus triggering apoptotic and inflammatory events.

To gain a mechanistic insight into how pDCs recognize HIV-infected cells, we used the Gen2.2 pDC-like cell line as a model [46]. We show that Gen2.2 cells, like primary pDCs, are sensitive to HIV replication and release IFN upon coculture with HIV-infected lymphocytes. We generated TLR7-negative Gen2.2 cells by RNA silencing. These cells were impaired in IFN production when cocultivated with HIV-infected lymphocytes, whereas CpG-induced TLR9 signaling was normal. These results provide a direct demonstration that TLR7 mediates recognition of HIV-infected cells. In addition, Gen2.2 cells recognized fusion-defective HIV significantly less potently than the wild-type virus, confirming that sensing of HIV-infected cells may occur at different intracellular locations in pDCs.

In this study, we also used 293T-derivatives as target cells. These non-hematopoïetic cells are not natural targets of HIV infection, but represent a valuable model to study HIV recognition in cells devoid of TLR7. In these targets, activation of the IFNβ promoter occurred upon coculture with HIV-infected lymphocytes, and not with free virions. Signaling required the expression of fusogenic envelope in donor cells, and the presence of cognate receptors (CD4 and CXCR4) in target cells. This strongly suggests that viral or cellular components originating from donor cells need to gain access to the cytoplasm of targets to activate the IFNβ promoter. We dissected further the signaling cascade leading to this activation. Promoter activation required the IRF3 transcription factor [55], as demonstrated by silencing this molecule. The role of IRF3 was confirmed by directly visualizing with the ImageStream apparatus its nuclear translocation, when target cells encounter HIV-infected cells. Of note, SeV activated more efficiently the IFNβ promoter than HIV-infected cells. This activation was particularly sensitive to IRF3 signaling (90% decrease in IFNβ promoter activity), and was associated with a marked nuclear translocation of IRF3. Thus, the stimulating effects of SeV and of HIV-infected cells on the IFN pathway are in part similar, rely on IRF3, but are not totally overlapping. That sensing of HIV-infected cells occurs via IRF3 signaling is in line with two recent reports, demonstrating that this transcription factor mediates recognition of HIV particles in different TLR7- cell types, including MDDCs and macrophages [32] [30].

Our observations raise intriguing questions about the nature of the cytosolic molecules recognizing HIV-infected cells. The cytosolic RLR (RIG-I-like receptors) helicases RIG-I and MDA5 may be involved in this phenomenon, since they directly recognize multiple and distinct forms of intracellular dsRNA. For instance, putative RIG-I ligands include 5-PPP-bearing RNAs, as well as RNAs with complex secondary structures [69], [70]. MDA5 binds to long dsRNAs [27], [71]. Addressing the role of RIG-I and MDA5 on recognition of HIV-infected cells will require further investigation. It will be also worth examining the implication of other cytosolic sensors that have been recently identified as mediating or modulating recognition of cell-free virions. This includes 3′ repair exonuclease 1 (TREX1) [72] and cyclophilin [32]. TREX1 acts as a negative regulator of the IFN response to DNA species generated during reverse transcription of endogenous retroelements [72]. TREX1 degrades HIV DNA and has been proposed to be used by the virus to avoid triggering detection by DNA sensors [30]. Cyclophilin binding to newly synthesized HIV Gag proteins also triggers an antiviral state in MDDCs [32]. It will be worth determining the role of TRIM5 molecules that bind incoming HIV capsids, regulate viral uncoating and thus exposure of HIV nucleic acids in infected cells. Whatever the underlying mechanism, our results demonstrate the existence of TLR7-independent pathways involved in the innate host response against HIV-infected cells.

What may be the relevance of this TLR7-independent signaling pathway? MDDCs are poorly responsive to cell-free HIV: they do not mature nor produce IFN when exposed to cell-free viral particles [14]. However, circumventing the restriction of MDDC to infection significantly enhances viral detection [32]. This innate response is dependent on cyclophilin A and subsequent IRF3 activation [32]. It will be worth determining if HIV-infected cells are sensed by MDDCs more potently than free virions, and if so, examine the role of Gag- cyclophilin interactions in this process.

In sum, our results show that the innate immune system efficiently senses HIV-infected lymphocytes. The potent recognition of HIV-infected cells may overcome the poor detection of cell-free virions. Various cellular sensors are involved in this recognition, which likely represents a driving force regulating the pathology of infection, as well as the generation of an adaptative immune response. How do the different sensor pathways interact to coordinate the host response to viral infection remains an outstanding question. Deciphering the basic mechanisms of HIV recognition has obviously important implications for the development of tailored vaccine strategies.

## Materials and Methods

### Cells, viruses and reagents

PBMCs were isolated from the blood of healthy donors by Ficoll centrifugation. The blood was provided by the EFS (Etablissement Français du Sang, the French Official Blood Bank). pDCs were isolated by positive selection using anti-BDCA-4 magnetic beads (Miltenyi Biotec). The negative fraction was collected and constituted PBMCs depleted from pDCs. CD4+ T lymphocytes were isolated from PBMCs by positive selection using magnetic beads (Miltenyi Biotec). CD4+ T lymphocytes were activated by PHA and grown with rhIL-2 for 3–5 days before infection. MT4C5, Jurkat T cells, PBMCs, and primary lymphocytes were grown in RPMI medium with 10% heat-inactivated fetal bovine serum (FBS). Monocyte-derived DCs (MDDCs) were prepared using GMCSF and IL-13-containing medium as described [73]. Isolation procedures yielded immature DC (CD1a+, MHC-I+, MHC-II+, CD64-, CD83-, CD80-low, CD86-low cells). HeLa, 293T and derivatives were grown in DMEM supplemented with 10% FBS. 293T-4X4 and 293T CXCR4 were generated using lentiviral transduction of 293T with lentiviruses coding for CD4 and/or CXCR4 molecules and magnetic sorting (Miltenyi Biotec). P4C5 are HeLa-CD4+ CCR5+ cells expressing a HIV-LTR-βGalactosidase reporter cassette. The pDC cell line Gen2.2 has been described elsewere [46]. Gen2.2 cells were grown in RPMI medium supplemented with 10% FBS, 1 mM sodium pyruvate and non-essential amino acids. Hela-Env cells stably express gp120/gp41 at the surface [45].

40 UHA/mL Influenza virus (FLUAV, A/PR/8/34, Charles River Laboratories) was used to stimulate PBMCs, Gen2.2 and 293T-4X4. Sendai virus (SeV, Cantell strain, ATCC, VR-907 Parainfluenza 1) was kindly provided by Dominique Garcin [54] and 4 UHA/mL were used to stimulate 293T-4X4. The production and use of HIV strains, including NL4-3 and NLAD8, NLΔEnv and NLF522Y mutants, and primary isolates BON and 132W [38] have been described [42], [65]. Viruses were either produced by transfection of 293T cells or by infection of MT4C5 cells. NLF522Y provirus encodes a non-fusogenic gp120/g41 Env complex [65]. Vesicular stomatitis virus type G (VSV-G) pseudotyped viruses NLΔEnv and NLF522Y were generated by cotransfection of 293T cells with the corresponding proviruses and a VSV-G expression plasmid.

### Mutant proviruses

The Reverse Transcriptase defective virus (RT-) was designed by replacing to D residues that form the catalytic site, DD185-186AS [74]. The protease negative mutant includes a D25E mutation in the enzyme. The Integrase mutants C130G (IN-) has been described [75]. The RnaseH mutant E478Q (RH-) and Nucleocapsid (NC44) have been described previously [74] [44].

Nevirapine (NVP, at 25 µM) is an inhibitor of HIV reverse transcriptase provided by the NIH AIDS Research and Reference Reagent Program. A151 (5 µg/mL), a TLR7 antagonist [43], was synthesized by the Institut Pasteur Genopole. Bafilomycin A1 (125 nM) was purchased from Sigma-Aldrich. CpG-2216 (Invivogen) was used at 5 µM.

siRNAs (anti-IRF3, sequence TCTGGATGAGTTACTGGGTAA ref SI03117359, and CTRL, sequence AATTCTCCGAACGTGTCACGT ref SI03650325) were from Qiagen, and were transfected according to the manufacturer with Hyperfect reagent.

### HIV infection, provirus transfection and cocultures

Two days prior to coculture, MT4C5 or activated primary CD4 T cells were infected with the indicated virus for 3 h at 37°C in the presence of 2 µg/mL DEAE Dextran (Sigma-Aldrich). MT4C5 cells expressing NLΔEnv and NLF522Y mutants, were obtained by infection with VSV-G pseudotyped viruses. The levels of living cells and of infected cells were assessed by cytometry analysis after HIV Gag staining. To distinguish donor or target cells, one of the population was stained with CarboxyFluorescein Diacetate Succinimidyl Ester (CFSE, 2.5 µM; Molecular Probes) for 10 mn at 37°C. Donor and target cells were then mixed at a ratio of 1∶2 or 1∶5 (PBMCs, PBMC-pDCs, Gen2.2) or 2∶1 (pDCs) in 96-well plate at a final concentration of 0.75×10^6^/mL (for PBMCs, PBMC-pDCs, Gen2.2), and 0.35×10^6^/mL (for pDCs) in a final volume of 250 µL. When stated, the indicated inhibitors were added to target cells 1 h before and maintained during the coculture.

HeLa cells (2×10^4^ cells per well, in 48-well plates) were transfected with the indicated HIV proviral vectors (1 µg) by lipofection (Roti®-Fect PLUS, Carl Roth GmbH) following manufacturer's instructions. One day later, CFSE-stained PBMCs were added at a concentration of 0.5×10^6^/mL. The infectivity of virions released in the supernatant of transfected HeLa cells was assessed using P4C5 cells [49]. Gen2.2 (10^6^ cells/well) were infected with the indicated doses of viruses, and HIV replication was assessed by following the appearance of Gag+ cells by flow cytometry and by measuring Gag-p24 release in supernatants by ELISA.

### Surface and intracellular staining

Cell surface stainings were performed at 4°C for 30 mn using anti-BDCA2-APC, anti-BDCA4-PE (Miltenyi Biotech), anti-CD4-PE (SK3, BD-Pharmingen), uncoupled anti-CCR5 (2D7, BD Pharmingen) and anti CXCR4 (12G5, NIH AIDS Research and Reference Reagent Program), followed by goat anti-mouse Ig (H+L)-PE (Southern Biotechnologies). Cells were intracellularly stained with anti-HIV-Gag (KC57-PE, Beckman-Coulter) and anti-MxA (a kind gift of Dr. O. Haller). Briefly, cells were fixed for 10 mn with PBS 4 % paraformaldehyde, washed, permeabilized and stained for 45 min in PBS containing 1 % BSA and 0.05 % saponin. Isotype-matched mAbs were used as negative controls. Samples were analyzed by flow cytometry using a FacsCalibur (Becton Dickinson) or a FacsCanto II (Becton Dickinson) with FlowJo or FacsDIVA softwares.

### Confocal imaging

For conjugates analysis, HIV-infected MT4C5 cells were mixed with CFSE-labelled Gen2.2 cells at a 1∶1 ratio and loaded on polylysine-coated coverslips (6×10^5^ cells in 400 µl). After 1 h at 37°C, cells were fixed and stained with an anti-Gag p24 and analyzed by confocal microscopy as described [76].

### Lentiviral transduction

Gen2.2 were transduced using lentiviral vector (LV) particles containing shRNA targeting TLR7 (NM_016562) Clone ID: TRCN0000056974 (OpenBiosystem) or control shRNA. The BVDV-NPro coding lentiviral vector has been described [77]. 293T-4X4 cells were transduced with a LV expressing BVDV-Npro.

The LV express also the puroR gene. Two days after transduction, Gen2.2 or 293T-4X4 cells were selected in the presence of 1 µg/mL puromycin. Resistant populations grew in a few days, and were used without further subcloning.

### Isolation of RNA and RT-PCR

Total RNA from Gen2.2 and 293T-4X4 cells were extracted using RNeasy Mini Kit (Qiagen), and cDNA was synthesized from 1 µg RNA using oligo-dT (Roche) and Superscript reverse transcriptase (Invitrogen) according to the manufacturers' instructions. Quantitative real-time PCR was performed as described [78] using the TaqMan gene expression assays technology (Applied Biosystems) for TLR7 (Hs00152971_m1), RIG-I (Hs00204833_m1), and MDA5 (Hs01070329_m1). GAPDH was used as a housekeeping gene to normalize mRNA expression. The ratio of gene of interest versus housekeeping gene was calculated according to the following formula: ratio  = 2^−dCt^ (dCT  =  mean Ct gene − mean Ct housekeeping). The mRNA relative level was calculated as the ratio between mRNA in transduced cells and mRNA in shCTRL cells. The reactions were run on a PTC200 equipped with a Chromo4 detector and analyses were performed with Opticon Monitor software version 2.03 (MJ Research). All the measures were performed in duplicate and validated when the difference in threshold cycle (Ct) between the 2 measures was <0.3.

### IFN detection

IFN secretion was quantified using the reporter cell line HL116, that carries the luciferase gene under the control of the IFN-inducible 6-16 promoter (a kind gift from Sandra Pellegrini, Institut Pasteur, France) [79]. HL116 were grown in DMEM supplemented with 10% FBS and HAT (H: 20 µg/mL, T: 20 µg/mL, A: 0.2 µg/mL). 2×10^4^ HL116 cells, plated 16 h prior the assay in 96-well plate, were incubated for 7 h with the desired culture supernatants or standards containing a titration of human IFNα2a (Immunotools). Cells were then lysed (Luciferase Cell Culture Lysis, 5X Reagent, Promega) and luciferase activity measured using the Luciferase Assay Reagent (Promega). Samples were analyzed using Perkin Elmer Wallac 1420. IFN levels are expressed as equivalent of IFNα2a concentration, in Unit/mL.

### Transient transfection and reporter gene assay

293T, 293T CXCR4 or 293T-4X4 cells were transfected using FuGENE 6 (Roche Diagnostics) according to the manufacturer's instructions. Cells were transiently cotransfected with 100 ng of IFNβ promoter-luciferase (generously provided by Dr. R. Weil and J. Hiscott), 20 ng of pRSV-β-galactosidase to control DNA uptake and expression. After 24 h, cells were exposed to SeV or or cocultured with HIV-infected MT4C5 at a ratio 1∶1 for 16 h. Cells were processed as previously reported [80]. Results are expressed as relative luciferase units (RLU) normalized with β-galactosidase activity. For silencing experiments, cell were transfected with siRNAs and were processed 48 h later as described above.

### Data and statistical analysis

Results of experiments are expressed as mean+SD. Comparisons between groups were performed using the Kruskal-Wallis test. Differences with a p-value of less than 0.05 were considered statistically significant.

## Supporting Information

Figure S1 a. Flow cytometry profile of PBMCs, PBMCs depeleted from pDCs, and enriched pDCs.
Cells are stained for BDCA4 and BDCA2 expression. The % of double positive cells in the circles are indicated. Results from one donor are representative of at least 5 independent donors. b. Expression of the IFN-inducible MXA protein by pDCs. pDCs were either left unstimulated (alone), exposed to the indicated amounts of cell-free HIV, to FLU, or coultivated with MT4C5 cells, either non infected (NI) or infected at two MOIs (high or low). 24h later, cells were stained for MXA and analyzed by flow cytometry. pDCs and MT4C5 cells were distinguished according to their SSC-H profile (X-axis). Results from one donor are representative of 3 independent donors.
(0.67 MB TIF)

Figure S2 HIV replication and IFN production in PBMCs.
PBMCs were activated with PHA and cultivated with IL-2, and 3 days later were exposed to HIV particles, NL4-3 strain, (10 ng/0.1 ml Gag p24/106 cells). Viral replication was assessed by following the appearance of Gag+ cells (left panel). IFN production was measured in supernatants (right panel). Data are representative of 3 independent experiments.
(0.18 MB TIF)
