# Supplementary figures and images for: Innate Sensing of HIV-Infected Cells
Source: PLoS Pathog. 2011 Feb 17;7(2):e1001284. doi: 10.1371/journal.ppat.1001284 (PMC3040675; doi:10.1371/journal.ppat.1001284)

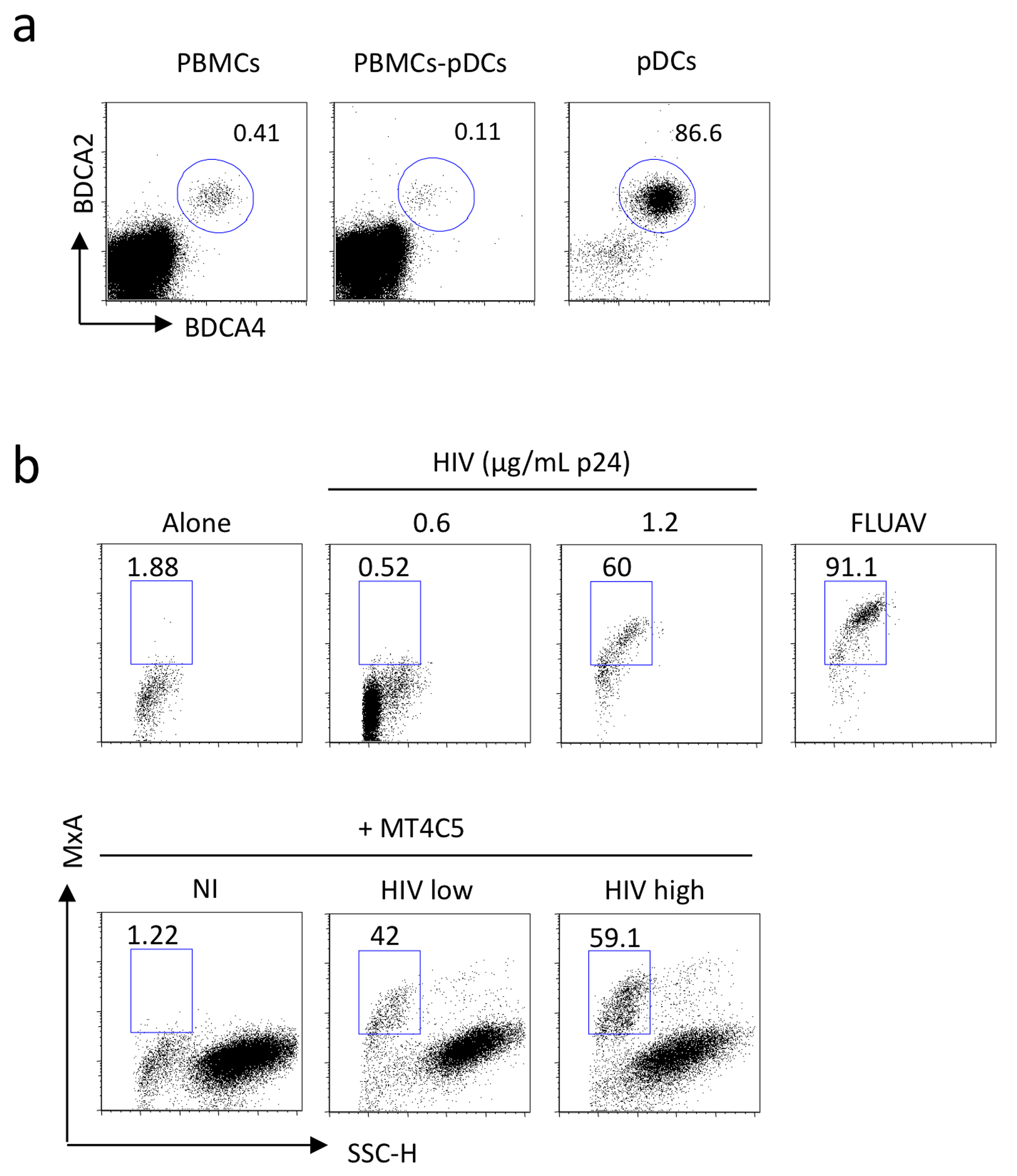

Supplement: Figure S1 — a. Flow cytometry profile of PBMCs, PBMCs depeleted from pDCs, and enriched pDCs. Cells are stained for BDCA4 and BDCA2 expression. The % of double positive cells in the circles are indicated. Results from one donor are representative of at least 5 independent donors. b. Expression of the IFN-inducible MXA protein by pDCs. pDCs were either left unstimulated (alone), exposed to the indicated amounts of cell-free HIV, to FLU, or coultivated with MT4C5 cells, either non infected (NI) or infected at two MOIs (high or low). 24h later, cells were stained for MXA and analyzed by flow cytometry. pDCs and MT4C5 cells were distinguished according to their SSC-H profile (X-axis). Results from one donor are representative of 3 independent donors. (0.67 MB TIF) [file ppat.1001284.s001.tif]

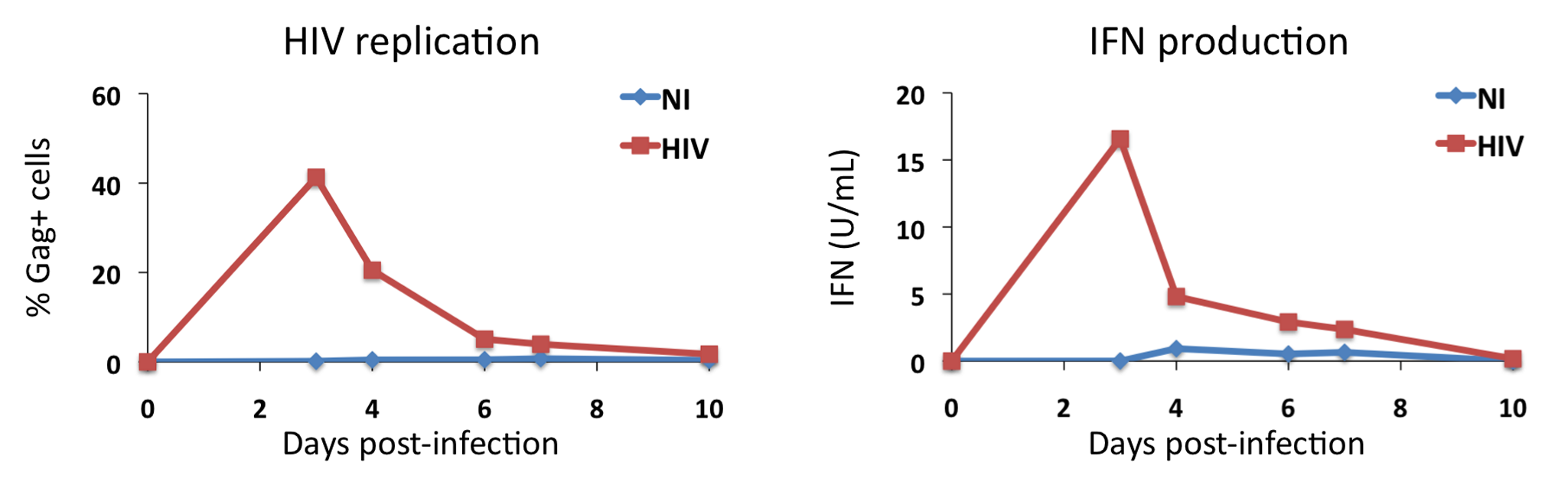

Supplement: Figure S2 — HIV replication and IFN production in PBMCs. PBMCs were activated with PHA and cultivated with IL-2, and 3 days later were exposed to HIV particles, NL4-3 strain, (10 ng/0.1 ml Gag p24/106 cells). Viral replication was assessed by following the appearance of Gag+ cells (left panel). IFN production was measured in supernatants (right panel). Data are representative of 3 independent experiments. (0.18 MB TIF) [file ppat.1001284.s002.tif]
